# Supplementary material for: A single N‐terminal phosphomimic disrupts TDP‐43 polymerization, phase separation, and RNA splicing
Source: EMBO J. 2018 Feb 9;37(5):e97452. doi: 10.15252/embj.201797452 (PMC5830921; doi:10.15252/embj.201797452)
Supplement: Supplementary file 1 — Appendix [file EMBJ-37-e97452-s001.pdf]

Appendix Figures

A single N-terminal phosphomimic disrupts TDP-43 polymerization, phase separation and RNA splicing

Ailin Wang*[1], Alexander E. Conicella[2]*, Hermann Broder Schmidt[3], Erik W. Martin[4], Shannon N Rhoads[5], Ashley N. Reeb[7], Amanda Nourse[4], Daniel Ramirez Montero[1], Veronica H. Ryan[6], Rajat Rohatgi[3,8], Frank Shewmaker[5], Mandar T. Naik[1], Tanja Mittag[4], Yuna M. Ayala[7], Nicolas L. Fawzi[1,2]

*AW and AEC contributed equally
Corresponding author: Nicolas L. Fawzi, Box G-E, Providence, Rhode Island 02912, email: Nicolas_Fawzi@brown.edu

[1]Department of Molecular Pharmacology, Physiology, and Biotechnology, and [2]Graduate Program in Molecular Biology, Cell Biology and Biochemistry, and [6]Neuroscience Graduate Program, Brown University, Providence, Rhode Island 02912, United States
[3]Department of Biochemistry, Stanford University School of Medicine, Stanford, CA 94305, United States
[4]Department of Structural Biology, St. Jude Children's Research Hospital, 263 Danny Thomas Place, Memphis, Tennessee, United States
[5]Department of Pharmacology and Molecular Therapeutics, Uniformed Services University, Bethesda, MD, United States
[7]Edward Doisy Department of Biochemistry and Molecular Biology, Saint Louis University School of Medicine, St. Louis, Missouri 63104, United States of America
[8]Department of Medicine, Stanford University School of Medicine, Stanford, CA 94305, USA

Keywords: ribonucleoprotein granule, amyotrophic lateral sclerosis, frontotemporal dementia, serine phosphorylation, biomolecular NMR spectroscopy, LLPS

Running title: TDP-43 NTD polymerization regulates LLPS/function

**Table of Contents**	**Page number**
Appendix Figures	2

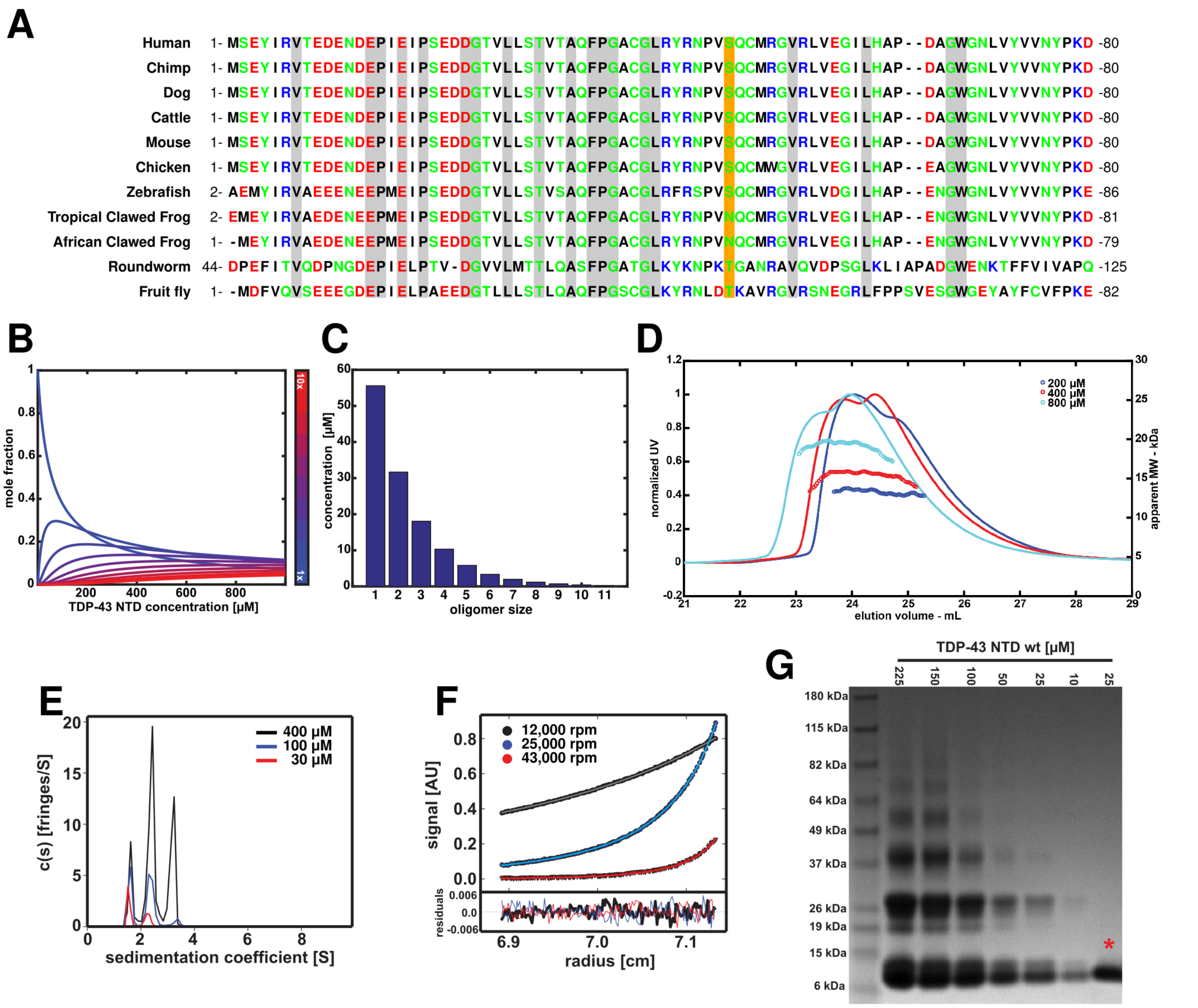

Appendix Figure S1: Data supporting NTD sequence conservation and isodesmic assembly

A Sequence alignment of the TDP-43 NTD across 11 species. Residues with strict conservation are highlighted in gray. S48 is largely conserved (S or T in all except *Xenopus* Species) and highlighted in orange.

B Mole fraction of monomeric (blue) through decameric (red) wild-type TDP-43 NTD species calculated for an isodesmic assembly as a function of total TDP-43 NTD concentration (x-axis) with K_D of 95 μM shows that monomer and low molecular weight species (cool colors) remain more populated (large magnitude) than long species through even up to 1 mM concentration.

C Concentration of TDP-43 species (in units of each multimer, not in units of monomers) for 300 μM wild-type TDP-43 NTD. In other words, this is a cross section of the data in (**B**) with multimer species concentration units.

D SEC-MALS profiles of wild-type TDP-43 NTD in 150 mM NaCl showing increasing apparent molecular weight as a function of injection concentration, consistent with weak (μM range) affinity.

E Data from sedimentation velocity AUC experiments show that the solution contains at least three species. Measurements were taken at three concentrations, 400, 100, 30 μM (black, blue, and red respectively).

F Sedimentation equilibrium AUC experiments were performed at protein concentration of 98 μM at three speeds 12,000 (for 36 hours) (black), 25,000 (for 24 hours) (blue) and 43,000 rpm (for 24 hours) (red). An isodesmic association model for self-assembly (solid lines) fit the experimental data well at all speeds with K_D of 40 μM.

G SDS-PAGE readout of TDP-43 NTD cross-linking assay as a function of concentration showing high molecular weight bands at higher NTD concentrations. Negative control (red star) in the absence of cross-linking agent.

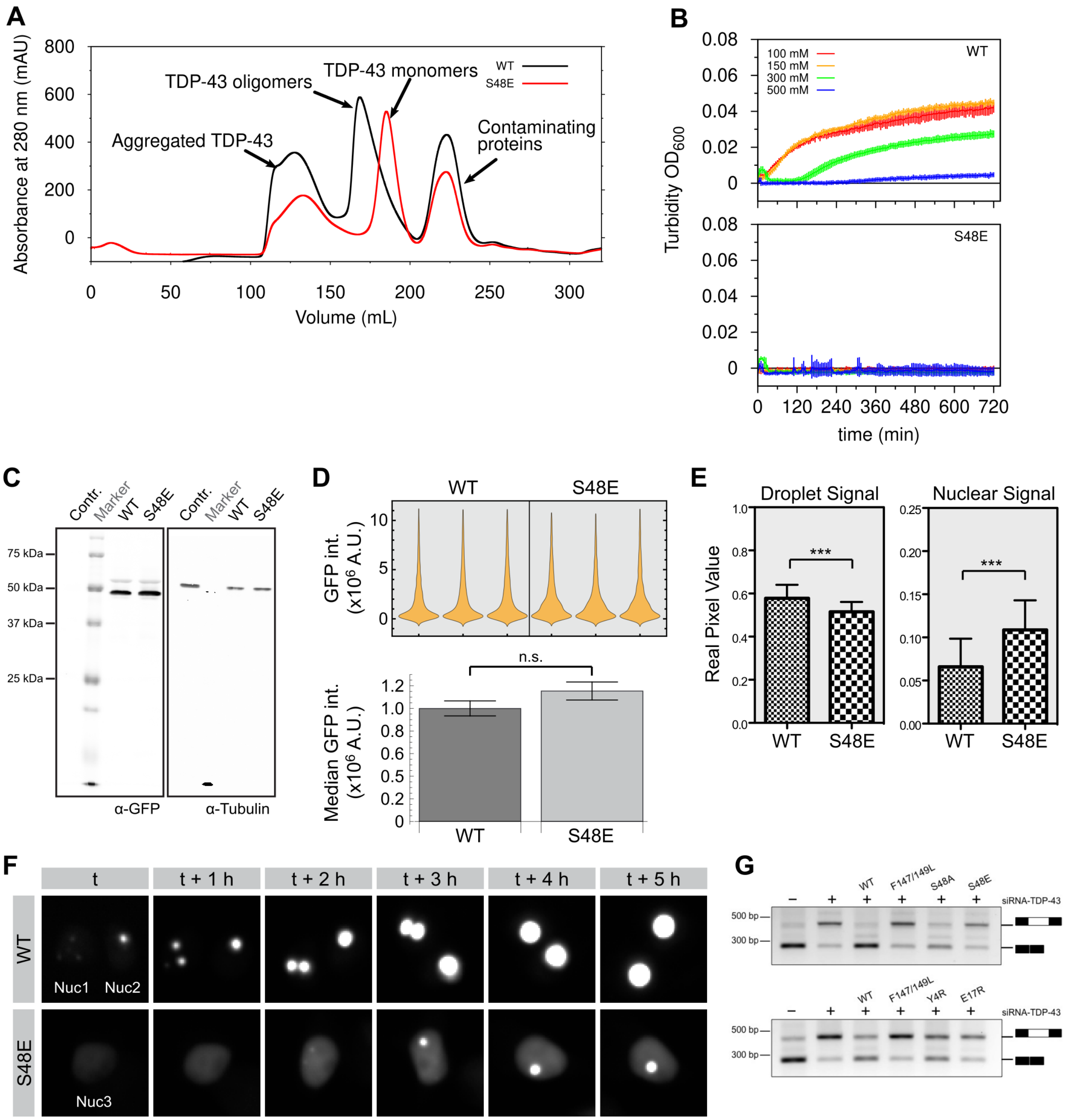

Appendix Figure S2: TDP-43 full length purification, phase separation, and expression in HeLa cells for splicing assays

A Superdex 200 26/600 size exclusion chromatogram of HisTrap HP purified full-length wild-type (black) and S48E (red) TDP-43 fused to a C-terminal GFP. Although some portion of aggregated protein is evident in the void and low molecular weight contaminants at long elution times, monomeric TDP-43 is purified from S48E. Absent in S48E TDP-43-MBP, an equilibrium of higher molecular weight wild-type TDP-43-MBP structures mediated by NTD interactions is suggested by the high molecular weight for wild-type and the skewed peak shape, as observed for TDP-43 NTD alone (Figure 1A).

B Turbidity vs. time as a function of NaCl concentration after addition of TEV to full-length TDP-43-MBP (as presented in Figure 2A, B). For wild-type (top), cleavage of the MBP results in

initiation of initial LLPS followed by conversion to aggregates or gels (Figure 2B) at long times. For S48E (bottom), no LLPS or aggregation is observed by turbidity.

C Determination of total WT and S48E TDP-$43_{RRM-GFP}$ reporter expression levels in 293T cells by immunoblotting. Full anti-GFP and anti-alpha-Tubulin immunoblots corresponding to Figure 2D with molecular weight markers.

D Determination of total WT and S48E TDP-$43_{RRM-GFP}$ reporter expression levels in 293T cells by flow cytometry. Above: Violin plot showing GFP intensities in cells for three independent transfections per reporter. Below: Bar chart comparing the median GFP intensities of the three WT and S48E populations. Statistical analysis (unpaired t-test) revealed no significant differences ($p = 0.0628$).

E Quantification of droplet (recorded with standard detector settings) and nuclear (recorded with sensitive detector settings) GFP signals for WT and S48E reporters. N > 80 cells were analyzed for both WT and S48E. Both signal differences are significant according to unpaired t-tests ($p < 0.0001$).

F WT and S48E reporter phase formation after transfection over time. Point t was defined as the first time point after transfection at which a GFP signal was observed in a given cell. Typically, this corresponds to 10-12 hours after transfection for both WT and S48E. In the representative images shown for WT and S483, two (Nuc1 and Nuc2) and one (Nuc3) nuclei are present, respectively.

G Representative agarose gel electrophoresis showing the products from the cellular minigene reporter with CFTR exon 9 inclusion and exclusion. Percent exon inclusion is quantified for each sample of control and siRNA treated HeLa cells plus siRNA-resistant wild-type (WT) and mutant TDP-43 constructs. A non-targeting siRNA was used as control.

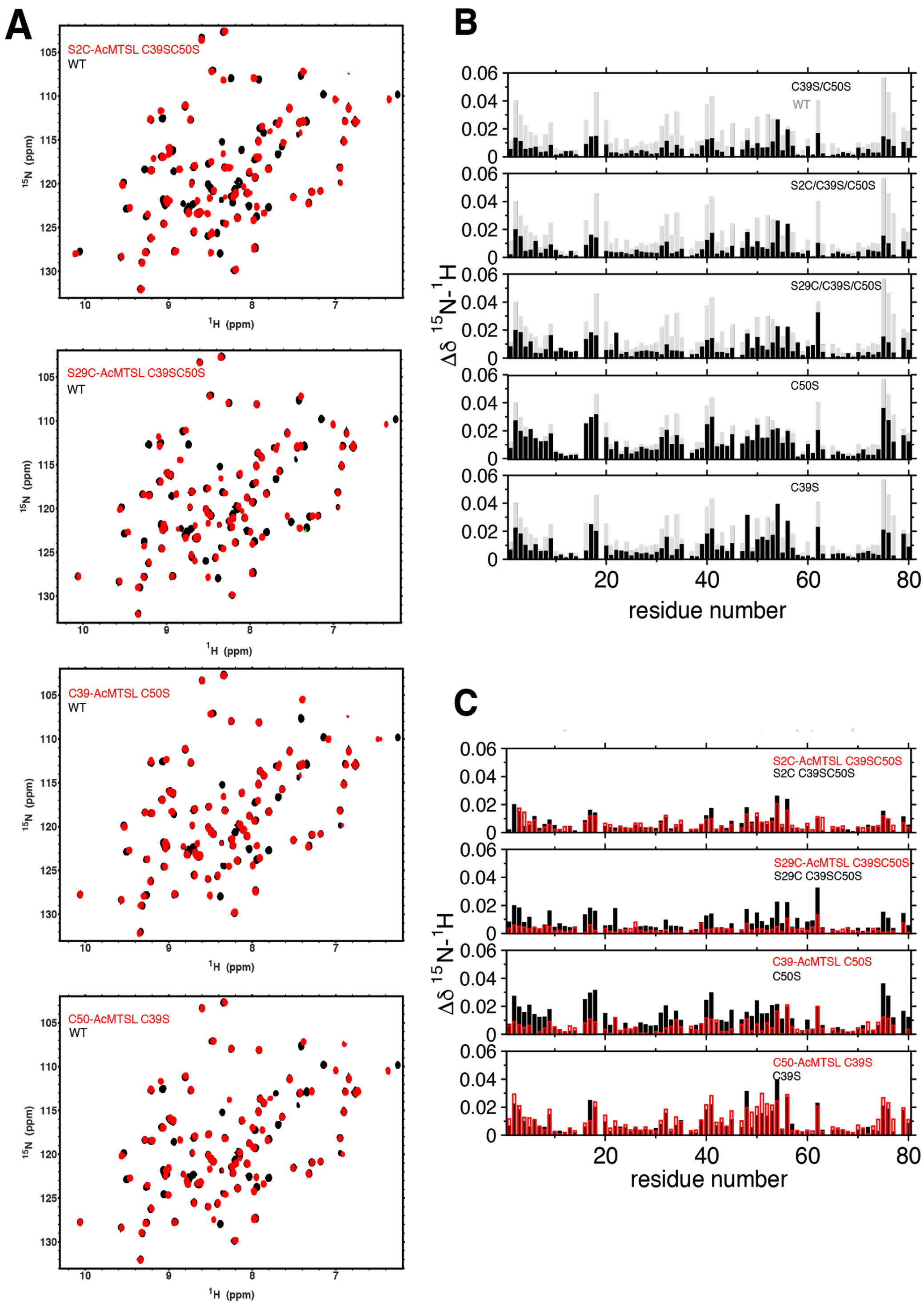

Appendix Figure S3: PRE mutation and spin-labeling have no significant effects on the structure and assembly. A Overlay of ^{15}N-^{1}H HSQC spectra of AcMTSL-labeled variants and wild-type show no large structural changes. **B** The CSDs at 100 μM compared to at 5 μM reference samples of each PRE variant (black) show similar profiles but reduced shifts compared to WT (grey), consistent with weaker binding. **C** The CSDs of AcMTSL-labeled PRE variants (red open) compared to the respective unlabeled variants (black) measured at 100 μM.

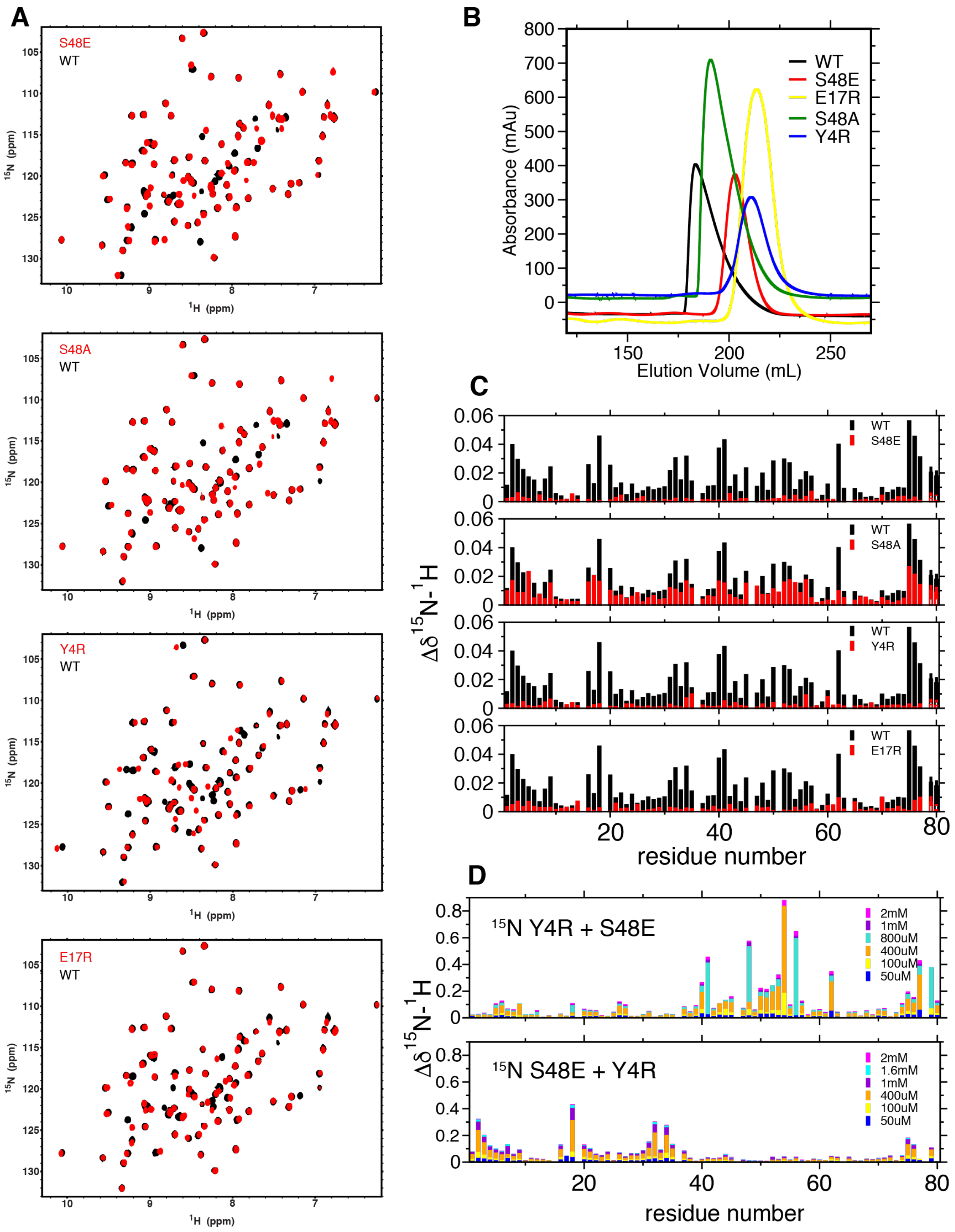

Appendix Figure S4: Interface-disrupting variants

A Overlay of HSQC spectra of variants (red) and WT (black) show no global structural differences. **B** Superdex 75 26/600 size exclusion chromatogram of WT and assembly disrupting variants. The baseline was not corrected. **C** CSDs of variants (red) compared to WT (black) measured at 100 μM, show decreased binding for S48E, Y4R, E17R as well as slight reduction for S48A. **D** The CSDs of titration Y4R-S48E are consistent with two interfaces.

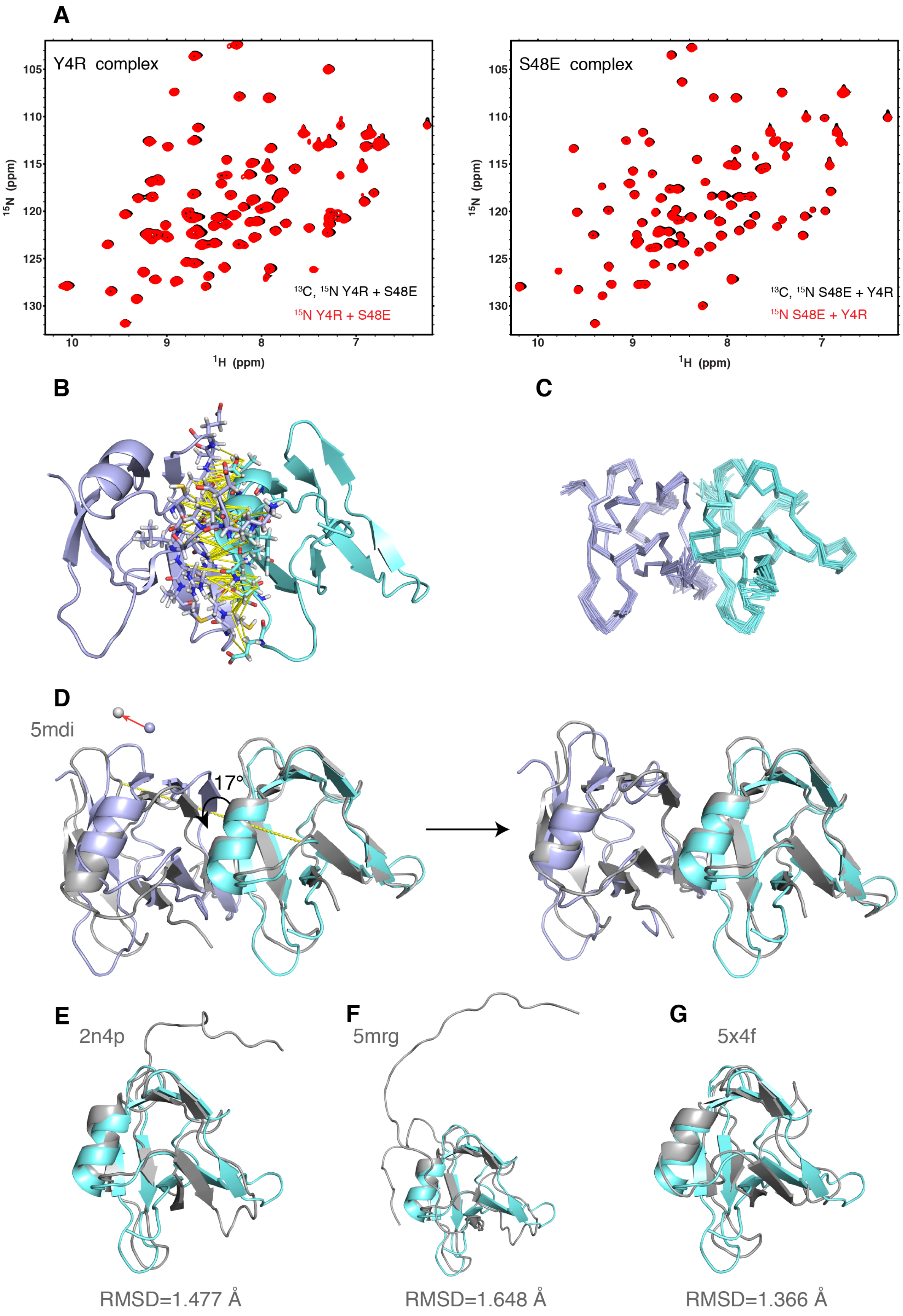
A
Y4R complex
S48E complex
^{13}C, ^{15}N Y4R + S48E
^{15}N Y4R + S48E
^{13}C, ^{15}N S48E + Y4R
^{15}N S48E + Y4R
^{15}N (ppm)
^{1}H (ppm)
B
C
D
5mdi
17°
E
2n4p
RMSD=1.477 Å
F
5mrg
RMSD=1.648 Å
G
5x4f
RMSD=1.366 Å

Appendix Figure S5: Construction of dimeric complex by mixing Y4R and S48E variants

A The overlaid HSQC spectra of filtered NOE sample (black) of 700 μM ^{13}C, ^{15}N-labedled Y4R (or S48E) and 2 mM natural abundance S48E (or Y4R), and the titration sample (red, copy from Figure 4A) of 100 μM ^{15}N-labeled Y4R (or S48E) and 2 mM natural abundance S48E (or Y4R) in which the formation of dimer is saturated. The consistent chemical shift confirms that the filtered NOE were collected from a sample with a dimer population nearing saturation. **B** The complete intermolecular NOEs are depicted as yellow lines (PDB ID 6B1G, Y4R in blue and S48E in cyan). The involved residues are shown in sticks. **C** Superimposition of the backbone ribbons of 20 lowest energy NMR ensembles. **D** Superimposition of the NMR dimeric structure (6B1G) in which S48E (cyan) is aligned with the chain B of crystal structure (5MDI, gray). To then align Y4R (blue) to crystal structure chain A, Y4R is rotated 17° about an axis through the center of mass of the other monomer (yellow dashed line) and translated according to the vector [-1.955 Å, 0.834 Å, 0.126 Å] (red arrow). **E-G** Superimposition of the previously published TDP-43 NTD monomer structures (gray) to the S48E (cyan). The Cα RMS deviation is calculated using model 1 in each NMR structural ensemble.

Appendix Table S1: NMR and refinement statistics for TDP-43 dimer structure

	**S48E**	**Y4R**
**NMR distance and dihedral constraints**		
Distance constraints		
Total NOE	1547	1459
Intra-residue	292	281
Inter-residue		
Sequential ($\lvert i-j \rvert = 1$)	472	458
Medium-range ($\lvert i-j \rvert < 4$)	256	243
Long-range ($\lvert i-j \rvert > 5$)	527	477
Intermolecular	116	116
Hydrogen bonds	48	46
Total dihedral angle restraints		
ϕ	61	59
ψ	61	59
**Structure statistics for complex**		
Violations		
Distance constraints (Å)	0.03	
Dihedral angle constraints (º)	0.54	
Max. dihedral angle violation (º)	5.90	
Max. distance constraint violation (Å)	0.65	
Average pairwise r.m.s. deviation (Å)		
Heavy	1.0	
Backbone	0.6	
Ramachandran analysis (%)		
Most favored regions	87.5	
Additional allowed regions	10.8	
Generously allowed regions	1.7	
Disallowed regions	0.0	

Table S1: Statistics for NMR solution structure of TDP-43 NTD S48E and Y4R dimer
